# Prognostic impact of C-reactive protein in elderly patients with acute heart failure and preserved ejection fraction: the modulating role of carbohydrate antigen 125

**DOI:** 10.3389/fcvm.2025.1611644

**Published:** 2025-08-15

**Authors:** Marina García, Pau Llàcer, François Croset, Jorge Campos, Carlos Pérez, Alberto Pérez, Marina Vergara, Paul Cevallos, Esteban Pérez, Cristina Fernández, María Pumares, Almudena Vázquez, Martín Fabregate, Luis Manzano

**Affiliations:** ^1^ Internal Medicine Department, Hospital Universitario Ramón y Cajal, IRYCIS, Madrid, Spain; ^2^ Department of Medicine and Medical Specialties, Facultad de Medicina y Ciencias de la Salud, Universidad de Alcalá, IRYCIS, Madrid, Spain

**Keywords:** C-reactive protein, carbohydrate antigen 125 (CA 125), acute heart failure (AHF), preserved, inflamation

## Abstract

**Aims:**

The systemic inflammation in heart failure (HF) is a common process, even more evident in acute scenario. Elevated C-reactive protein (CRP) is typically linked to increased morbidity and mortality in both acute and chronic heart failure. Moreover, Carbohydrate Antigen 125 (CA125) is elevated in most of the AHF patients. In this cohort of elderly patients admitted for AHF and preserved ejection fraction, our objective was to evaluate the association between CRP values and long-term outcomes, stratified by plasma CA125 concentration.

**Methods and results:**

This retrospective cohort study included 453 elderly patients hospitalized for acute heart failure with preserved ejection fraction. Patients were categorized into four groups based on CRP (>20 mg/dl) and CA125 (≥35 U/ml) levels. The primary endpoints were all-cause mortality and heart failure readmission. Median age was 87 years (IQR: 85–89), and 72.6% were women. During a median follow-up of 463 days, 358 patients (54.9%) died and 208 (45.9%) were rehospitalized. In multivariable Cox models, a significant interaction was observed between CRP and CA125 for mortality (*p* for interaction = 0.05). Patients with both elevated CRP and CA125 had the highest mortality risk (HR: 1.79, 95% CI: 1.27–2.10; *p* < 0.001), while CRP elevation alone was not associated with increased risk. A similar trend was observed for readmission (HR: 1.50, 95% CI: 1.07–2.11; *p* = 0.019), though the interaction did not reach significance (*p* = 0.080).

**Conclusion:**

In patients with acute heart failure and preserved ejection fraction, the prognostic impact of CRP is influenced by CA125 levels. High CRP levels were associated with higher risk of death or heart failure hospitalization only when coexisted with high CA125. On the contrary, when CA125 was low, high CRP lacked prognostic effect.

## Introduction

The systemic inflammation in heart failure (HF) is a common process observed especially in comorbidity older patients with preserved ejection fraction phenotype, even more evident in acute scenario (1–4). The role of inflammation in the pathophysiology of HF is linked with the comorbidities will induce systemic vascular inflammation, leading to endothelial dysfunction, myocardial fibrosis, high diastolic stiffness, and clinical HF (1–7).

C-reactive protein (CRP), synthesized by hepatocytes in response to IL-6 and IL-1 signalling, is a key marker of inflammation, infection or tissue damage (8, 9). Elevated CRP is typically linked to increased morbidity and mortality in both acute and chronic heart failure (10–12). However, in patients with AHF, most studies on the prognostic value of CRP have focused on those with reduced ejection fraction (HFrEF) (11–17).

Carbohydrate Antigen 125 (CA125) is a high molecular weight glycoprotein that is elevated in most AHF patients (18). Previous studies suggest that elevated CA125 in HF is due to at least two pathophysiological mechanisms (19, 20). Firstly, CA125 is associated with increased volume overload produced by mechanical stress due to excessive fluid accumulation (19). Secondly, high CA125 values are associated with worse prognosis, independent of previous volume overload or residual congestion. This fact is suggestive of its involvement in other pathophysiological processes such as underlying inflammation (19).

Previous evidence supports the association between CA125 with certain proinflammatory cytokines such as tumour necrosis factor (TNF)-α, interleukin (IL)-6, and IL-10 (17–21). Hence, we hypothesize that the combination of elevated CRP and CA125 levels identifies patients with persistent vascular permeability and significant extravascular fluid accumulation. As a result, the negative prognostic impact of high CRP levels in heart failure may be particularly pronounced when CA125 is also elevated, highlighting the interplay between inflammation and fluid retention.

In this cohort of elderly patients admitted for AHF and preserved ejection fraction, our objective was to evaluate the association between CRP values and long-term outcomes, stratified by plasma CA125 concentration.

## Material and methods

### Study design and population

This is a retrospective observational study of a cohort of 453 patients. This cohort included patients admitted to the Internal Medicine department of the Hospital Ramón y Cajal with the diagnosis of AHF and preserved ejection fraction.

AHF was defined as the rapid onset of symptoms and signs secondary to abnormal cardiac function and the presence of objective evidence of structural or functional abnormality of the heart rest, according to current guidelines (22). Demographic data, medical history, vital signs, 12-lead electrocardiogram, laboratory data and treatments were determined during hospitalization. Treatment with angiotensin converting enzyme inhibitors, angiotensin receptor blocker, mineral receptor antagonists, sodium-glucose co-transporter-2 inhibitors, beta blockers, furosemide and other therapeutic strategies were individualized following established guidelines (22). Patients whose main diagnosis was a severe infectious condition were excluded. CRP values greater than 100 mg/L were disregarded to reduce potential confounding by acute infections.

This study was carried out in accordance with the Declaration of Helsinki and was approved by the ethics committee of the Hospital Ramón y Cajal. All participants signed an informed consent form before participating in this study.

## Laboratory analysis

Blood tests were assessed on admission (at least 24 h after admission to the emergency) and analysed in the local laboratory of each participating site, including CRP plasma levels and CA125. We established a cut-off point of CA125 > 35 UI/ml as an abnormal value.

## Endpoints

Patient's follow-up was censored if death, or time with a median of two years. The endpoint of interest was time to all-cause mortality and heart failure hospitalization. Adverse clinical endpoints were verified through electronic patients' clinical charts and adjudicated by an investigator who was blinded to the patient's levels of both biomarkers.

## Statistical analysis

Continuous variables were expressed as median and interquartile ranges (IQR). Discrete variables were summarized as percentages. CRP and CA125 were dichotomized based on firstly, according to the median (CRP > 20 mg/dl) as in previous studies (13) and on established cut points cut-off (CA125 > 35 U/ml) (23). A variable with 4 categories was formed by combining these variables: C1 = CRP < 20 mg/dl and CA125 < 35 U/ml (*n* = 69); C2 = CRP > 20 mg/dl and CA125 < 35 U/ml (*n* = 158); C3 = CRP < 20 mg/dl and CA125 > 35 U/ml (*n* = 69); C4 = CRP > 20 mg/dl and CA125 > 35 U/ml (*n* = 157).

Comparisons across categories were performed by a *χ*^2^ test for categorical variables. For continuous variables, the appropriate ANOVA or Kruskal–Wallis was used for variables with a parametric and nonparametric distribution, respectively. All covariates shown in Table 2.

To test the working hypothesis, the relationship between CPR levels and clinical outcome was assessed separately in four categories based on groups of patients: those with low or high levels of CA125 (< or ≥35 U/ml, respectively). Survival analysis was performed using the Kaplan–Meier method and the log-rank test to compare the survival curves between patients according to the categories. Such analysis was carried out by a Cox proportional hazard regression, and *p*-value for interaction and estimates of risk attributable to CRP across CA125 strata were expressed as hazard ratios (HR) with 95% confidence intervals (CI). Model discrimination was assessed using Harrell's C-statistic (0.667 for all-cause mortality, 0.649 for rehospitalization).

Missing data were handled through complete-case analysis, as missingness was <5% for all variables. To confirm robustness, we also performed multiple imputation using chained equations (five imputations), which yielded consistent results. Model calibration was evaluated using calibration plots comparing predicted and observed survival at 1-year follow-up, demonstrating acceptable agreement. The proportional hazards assumption was tested using Schoenfeld residuals and global tests, with no violations observed (*p* > 0.10 for all variables).

We set a two-sided *p*-value of <0.05 as the threshold for statistical significance. Stata 18 (StataCorp. 2023. Stata Statistical Software: Release 18. College Station, TX: StataCorp LLC.) was used for these analyses.

## Results

The median (IQR) age was 87(85–89) years, 326 (72.6%) were women. The median (IQR) left ventricular ejection fraction (LVEF) were 61.6% (53–67.7). The most prevalent comorbidities were hypertension (89.6%), atrial fibrillation (AF) (60%), chronic kidney disease (CKD) (50%), and diabetes mellitus (DM) (37.9%). The median (IQR) values of CRP, CA125 and B-type Natriuretic Peptide (BNP) were 22.2 mg/dl (7.1–51), 58.2 (28–120.2) U/ml and 547.4 (305–991.5) pg/ml, respectively. The proportion of patients with CRP ≥20 mg/dl and CA125 ≥ 35 U/ml were 49.6% and 69.6%, respectively (Table 1).

**Table 1:** Baseline characteristics across CRP levels.

Variables	Total (*n* = 453)	Low CRP (*n* = 227)	High CRP (*n* = 226)	*p* value
Demographic parameters and medical history				
Age, years	87 (83–90)	87 (82–90)	87 (83–90)	0.636
Female sex. *n* (%)	298 (65.7)	155 (68.2)	143 (63.27)	0.261
Hypertension, *n* (%)	406 (89.6)	205 (90.3)	201 (88.9)	0.632
Diabetes mellitus, *n* (%)	172 (37.9)	99 (43.6)	73 (32.3)	0.013
COPD, *n* (%)	87 (19.2)	45 (19.8)	42 (18.5)	0.738
Atrial Fibrilation, *n* (%)	272 (60)	145 (63.8)	127 (56.1)	0.095
Ischemic heart disease, *n* (%)	114 (25.1)	52 (22.9)	62 (27.4)	0.267
Valvular disease, *n* (%)	150 (33.1)	73 (32.1)	77 (34)	0.665
Neoplasia, *n* (%)	51 (11.26)	20 (8.8)	31 (13.7)	0.09
Days of hospitalization, (d)	6 (4–8)	5 (4–7)	6 (4–9)	<0.001
Physical examination				
NYHA class (%)				0.836
I	17 (3.9)	8 (3.5)	9 8 (3.9)	
II	293 (67.3)	152 (66.9)	141 (62.3)	
III	121 (27.8)	57 (25.1)	64 (28.3)	
IV	4 (0.9)	2 (0.8)	2 (0.8)	
Systolic Blood Pressure, mmHg	136 (120–150)	138 (122–143)	135.5 (120–150)	0.317
Diastolic Blood Pressure, mmHg	72 (62–87)	74 (63–90)	70 (61–84)	0.091
Heart rate, bpm	80 (70–93)	80 (70–94)	80 (68–92)	0.482
Peripheral edema, *n* (%)	305 (67.3)	150 (66)	155 (68.5)	0.570
Echocardiogram				
LVEF, (%)	61.6 (53–67.7)	60.3 (52–67.6)	62.45 (54.6–68)	0.247
TAPSE, cm	1.9 (1.71–2.3)	1.9 (1.7–2.2)	2 (1.77–2.35)	0.043
sPAP, mmHg	46 (36.8–58.9)	46.8 (36–60)	45.2 (37.2–57.2)	0.653
Inferior vena cava, *n* (%)	79 (40.5)	40 (17.6)	39 (17.2)	0.747
Pulmonary B lines, *n* (%)	65 (33.3)	30 (13.2)	35 (15.4)	0.543
Pleural effusion, *n* (%)	199 (44)	92 (40.5)	107 (47.3)	0.144
Laboratory parameters				
Creatinine, mg/dl	1.2 (0.8–1.6)	1.2 (0.8–1.6)	1.3 (0.8–1.7)	0.676
eGFR, ml/min/1.73 m^2^	46.3 (33.5–64)	50 (35.5–62.9)	44.2 (30.5–64.4)	0.291
Urea, g/dl	63 (45–94)	64 (44–64)	63 (45–92.5)	0.957
Sodium, mEq/L	139 (136–142)	139 (136–142)	139 (135–142)	0.250
Potassium, mEq/L	4.5 (4.1–5)	4.5 (4.1–5)	4.5 (4.1–4.9)	0.353
Chloride, mEq/L	102 (98–106)	102 (99–107)	102 (98–106)	0.599
Hemoglobine, g/dl	12 (10.6–13.3)	12.4 (11–13.7)	11.7 (10.1–13.1)	<0.001
Leukocytes, mcg/L	6,790 (11–9,230)	6,590 (9.7–8,340)	7,030 (11.8–10,100)	0.036
BNP, pg/ml	547.3 (305–991.3)	909 (285.5–1,153.5)	744.7 (498–1,132)	0.513
CA125, U/ml	60.5 (28–126)	67 (26.9–131.6)	58 (30.1–120.2)	0.615
Total proteins, g/dl	6.2 (5.8–6.6)	6.2 (5.8–6.6)	6.2 (5.8–6.7)	0.737
Albumin, g/dl	3.1 (2.8–3.3)	3.2 (3–3.4)	2.9 (2.6–3.1)	<0.001
Total cholesterol, mg/dl	134 (112–157)	134 (114.5–155)	134 (110.5–160.5)	0.745
Treatment				
ACEI/ARB, *n* (%)	244 (53.7)	61 (26.8)	51 (22.5)	0.414
Betablokers, *n* (%)	262 (58.0)	142 (62.5)	120 (53)	0.104
SGLT2i, *n* (%)	35 (17.8)	18 (7.9)	17 (7.5)	0.933
MRA, *n* (%)	48 (10.6)	28 (12.3)	20 (8.8)	0.228
Furosemide, *n* (%)	310 (68.4)	161 (70.9)	149 (65.9)	0.253
Events				
Mortality, *n* (%)	172 (37.97)	72 (31.7)	100 (44.2)	0.006
Heart failure hospitalization, *n* (%)	208 (45.9)	97 (42.7)	111 (49.1)	0.173

COPD, chronic obstructive pulmonary disease; NYHA, New York heart association; LVEF, left ventricular ejection fraction; TAPSE, tricuspid annular plane systolic excursion; sPAP, systolic pulmonary artery pressure; eGFR, estimated Glomerular filtration rate, BNP, B-type natriuretic peptide; CA125, carbohydrate antigen 125; ACEI, angiotensin converting enzyme inhibitors; ARB, angiotensin receptor blocker; MRA, mineral receptor antagonists; SGLT2i, sodium-glucose co-transporter-2 inhibitors.

### Baseline profiles across CRP and CA125 categories

When we analyzed the sample based on CRP levels, we found that patients with high CRP had a demographic and echocardiographic profile largely like those with low CRP. The only notable differences were the lower prevalence of diabetes and a slightly higher TAPSE value among those with elevated CRP. However, these patients demonstrated a distinctly proinflammatory profile, characterized by lower hemoglobin and albumin levels, as well as higher leukocyte counts. Despite these differences, there were no significant variations in chronic treatment between the two groups (Table 1).

Furthermore, when the cohort was analyzed across the four categories based on CRP and CA125 levels, patients with elevations in both biomarkers exhibited the most severe clinical presentation. This subgroup had the longest hospital stays, a higher prevalence of peripheral edema, and significantly lower albumin and cholesterol levels. Most notably, this group also had the highest mortality rates (Table 2).

**Table 2:** Baseline characteristics.

Variables	Total (*n* = 453)	Categorie 1: Low CRP/LowCA125 (*n* = 69)	Categorie 2: High CRP/Low CA125 (*n* = 158)	Categorie 3: Low CRP/High CA125 (*n* = 69)	Categorie 4: High CRP/High CA125 (*n* = 157)	*p* value
Demographic parameters and medical history						
Age, years	87 (83–90)	86 (81–89)	87 (82–90)	86 (82–90)	87 (83–90)	0.798
Female sex. *n* (%)	298 (65.7)	53 (76.8)	102 (64.5)	46 (66.6)	97 (61.7)	0.157
Hypertension, *n* (%)	406 (89.6)	67 (97.1)	138 (87.3)	64 (92.7)	137 (87.2)	0.082
Diabetes mellitus, *n* (%)	172 (37.9)	33 (47.8)	66 (41.7)	20 (28.9)	53 (33.7)	0.061
COPD, *n* (%)	87 (19.2)	12 (17.3)	33 (20.8)	15 (21.7)	27 (17.1)	0.770
Atrial Fibrilation, *n* (%)	272 (60)	37 (53.6)	108 (68.3)	36 (52.1)	91 (57.9)	0.050
Ischemic heart disease, *n* (%)	114 (25.1)	11 (15.9)	41 (25.9)	20 (28.9)	42 (26.7)	0.271
Valvular disease, *n* (%)	150 (33.1)	23 (33.3)	50 (31.6)	19 (23.1)	58 (36.9)	0.539
Neoplasia, *n* (%)	51 (11.26)	8 (11.6)	12 (7.1)	10 (14.5)	21 (13.4)	0.313
Days of hospitalization, (d)	6 (4–8)	5 (3–6)	6 (4–8)	5 (4–7)	6 (5–11)	0.037
Physical examination						
NYHA class (%)						0.492
I	17 (3.9)	2 (2.8)	6 (3.7)	6 (8.6)	3 (1.9)	
II	293 (67.3)	49 (71.6)	103 (65.1)	42 (60.8)	99 (63)	
III	121 (27.8)	17 (24.6)	40 (25.3)	18 (26)	46 (29.2)	
IV	4 (0.9)	0 (0)	2 (1.2)	1 (1.4)	1 (0.6)	
Systolic Blood Pressure, mmHg	136 (120–150)	138 (118–150)	137.5 (122.5–155)	136 (122–150)	135 (120–147)	0.781
Diastolic Blood Pressure, mmHg	72 (62–87)	70 (62–82)	76 (63–91.5)	70 (60–83)	70 (61–85)	0.152
Heart rate, bpm	80 (70–93)	77 (72–92)	83 (70–95)	76 (66–87)	81 (70–93)	0.076
Peripheral edema, *n* (%)	305 (67.3)	48 (69.5)	102 (64.5)	39 (56.5)	116 (73.8)	0.059
Echocardiogram						
LVEF, (%)	61.6 (53–67.7)	61.6 (52.4–67.4)	60 (52–67.6)	62.8 (55.7–68)	62 (53–67.8)	0.428
TAPSE, cm	1.9 (1.71–2.3)	1.9 (1.8–2.2)	1.9 (1.7–2.2)	2 (1.8–2.4)	2 (1.7–2.3)	0.647
sPAP, mmHg	46 (36.8–58.9)	37.5 (32.4–46.7)	52.3 (43.7–60)	47 (40–56)	45 (37.1–57.8)	0.441
Inferior vena cava, *n* (%)	79 (40.5)	8 (11.5)	32 (20.2)	11 (15.9)	28 (17.8)	0.205
Pleural effusion, *n*, (%)	199 (44)	21 (30.4)	71 (45)	21 (30.4)	86 (55)	<0.001
Laboratory tests						
Creatinine, mg/dl	1.2 (0.8–1.6)	1.1 (0.8–1.5)	1.2 (0.9–1.7)	1.0 (0.8–1.7)	1.1 (0.9–1.8)	0.145
eGFR, ml/min/1.73 m^2^	46.3 (33.5–64)	58.2 (42.8–68.1)	46.8 (34.9–61.5)	45.1 (31.7–70.2)	43.9 (29.3–64)	0.449
Urea, g/dl	63 (45–94)	59 (44–90)	66 (43–94)	52 (40–90)	69.5 (47–99)	0.720
Sodium, mEq/L	139 (136–142)	139 (136–141)	139 (136–142)	138 (135–142)	139 (136–141)	0.583
Potassium, mEq/L	4.5 (4.1–5)	4.4 (4.1–5)	4.6 (4.2–5)	4.4 (4.1–4.8)	4.5 (4.1–4.9)	0.200
Chloride, mEq/L	102 (98–106)	102 (99–105)	102 (100–107)	102 (97–105)	102 (98–107)	0.163
Hemoglobine, g/dl	12 (10.6–13.3)	12.5 (11–13.7)	12.3 (11.1–13.7)	12.1 (10.6–13.3)	11.5 (9.9–12.9)	0.685
Leukocytes, mcg/L	6,790 (11–9,230)	7,000 (10.9–9,900)	6,405 (9.6–8,010)	7,190 (10.6–11,000)	6,690 (13.2–9,470)	0.135
BNP, pg/ml	547.3 (305–991.3)	350.8 (206.3–694.3)	722.2 (390–1,077)	424 (251.2–725.8)	620 (319–1,160.9)	<0.001
Total proteins, g/dl	6.2 (5.8–6.6)	6.4 (6–6.8)	6.1 (5.7–6.6)	6.4 (5.9–6.7)	6.1 (5.8–6.6)	0.560
Albumin, g/dl	3.1 (2.8–3.3)	3.2 (3.1–3.5)	3.1 (3–3.5)	3.1 (2.7–3.3)	2.9 (2.5–3.15)	<0.001
Total cholesterol, mg/dl	134 (112–157)	142 (118–171)	130 (112–151)	135 (120–165)	131.5 (108–158)	<0.001
Treatment						
ACEI/ARB, *n* (%)	244 (53.7)	42 (60.9)	88 (55.7)	39 (56.5)	75 (47.8)	0.249
Betablokers, *n* (%)	262 (58.0)	39 (56.5)	103 (65.1)	34 (49.2)	86 (54.7)	0.028
SGLT2i, *n* (%)	35 (17.8)	2 (2.8)	16 (10.1)	10 (14.4)	7 (4.4)	0.024
MRA, *n* (%)	48 (10.6)	11 (15.9)	17 (10.7)	4 (5.7)	16 (10.1)	0.285
Furosemide, *n* (%)	310 (68.4)	52 (75.3)	109 (68.9)	48 (69.5)	101 (64.3)	0.420
Events						
Mortality, *n* (%)	240 (53)	31 (44.9)	83 (52.5)	29 (42)	97 (61.8)	0.018
Heart failure hospitalization, *n* (%)	208 (45.9)	26 (37.7)	23 (33.3)	71 (44.9)	88 (56.1)	0.005

4 categories based on CRP and CA125 levels.

COPD, chronic obstructive pulmonary disease; NYHA, New York heart association; LVEF, left ventricular ejection fraction; TAPSE, tricuspid annular plane systolic excursion; sPAP, systolic pulmonary artery pressure; eGFR, estimated Glomerular filtration rate, BNP, B-type natriuretic peptide; CA125, carbohydrate antigen 125; ACEI, angiotensin converting enzyme inhibitors; ARB, angiotensin receptor blocker; MRA, mineral receptor antagonists; SGLT2i, sodium-glucose co-transporter-2 inhibitors.

### Association between the levels of CRP and CA125 categories and all-cause mortality

**Figure 1:**
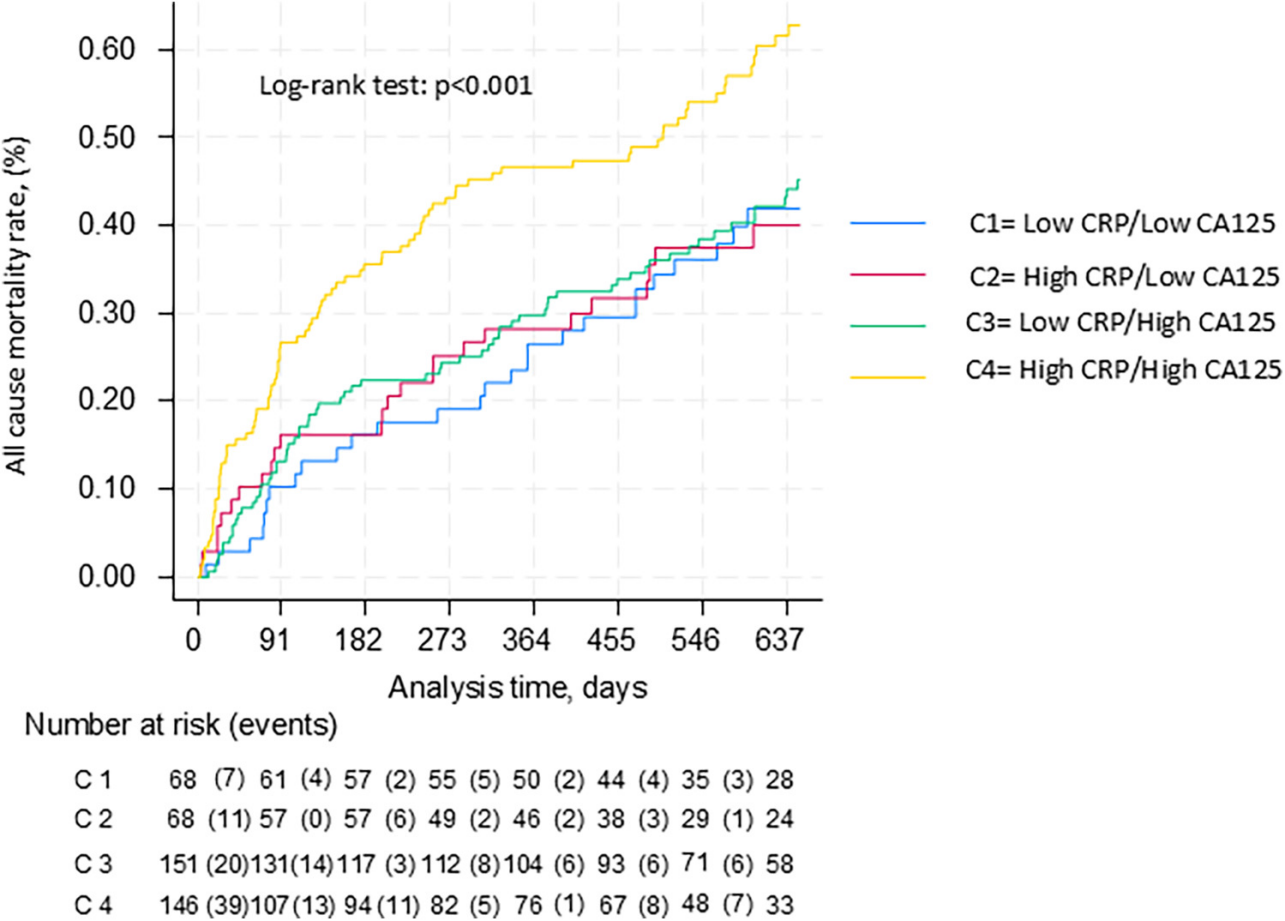
Kaplan-Meier curves between CRP and CA125 categories for all-cause mortality.

During a median (p25%–p75%) follow-up of 463 (116–695) days, 358 (54.9%) patients died. When stratifying the predictive value of CRP across CA125 ≤ 35 vs. >35 U/ml, we were able to reclassify the risk of patients (C1:43.3%, C2:54.3%, C3: 43.6%, and C4: 60.9%, *p* = <0.001). Kaplan–Meier plots showed divergent trajectories among categories, with a higher risk for patients with high CRP and high CA125, without overt differences for the other groups (Figure 1). This differential association of CRP, according to CA125, persisted significantly after multivariate adjustment (*p*-value for interaction = 0.05). Compared to the lower baseline risk group (CRP <20 mg/dl and CA125 <35 U/ml), patients with CRP ≥ 20 mg/dl and CA125≥ U/ml show the higher risk of adverse events (HR: 1.79, CI: 95% = 1.27–2.1, *p* < 0.001). Conversely, those with CRP ≥ 20 mg/dl and CA125 < 35 U/ml did not show a higher risk of adverse events (HR: 0.95, CI 95% = .55–1.64, *p* = 0.847).

### Association between CRP and CA125 categories and heart failure readmission

**Figure 2:**
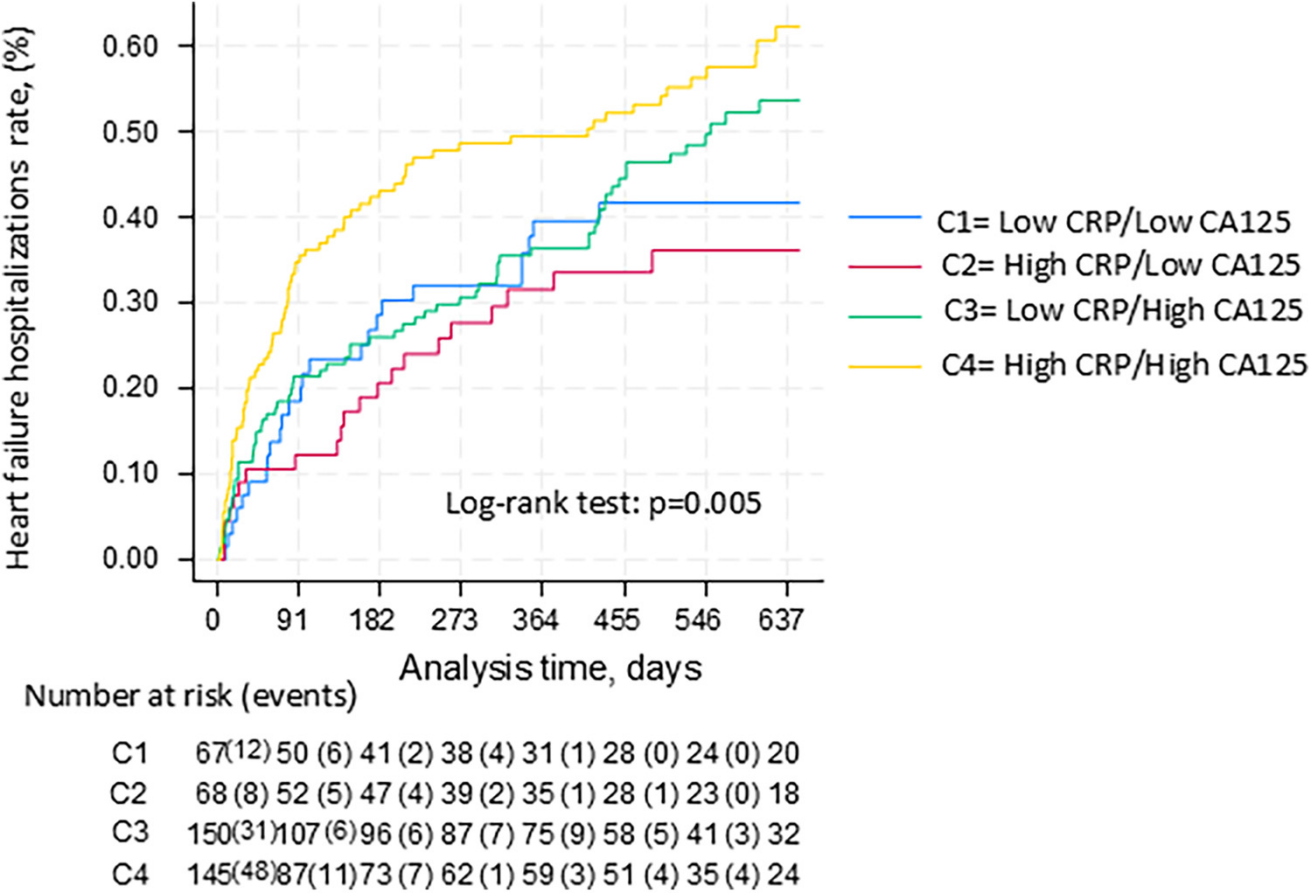
Kaplan-Meier curves between CRP and CA125 categories for heart failure hospitalizations.

During a median (p25%–p75%) follow-up of 463 (116–695) days, 208 (45.9%) patients were readmitted for HF. When stratifying the predictive value of CRP across CA125 ≤ 35 vs. >35 U/ml, we observed a differential risk pattern across categories (C1: 37.7%, C2: 33.3%, C3: 44.9%, and C4: 56.1%; log-rank test: *p* = 0.005). Kaplan–Meier curves showed significantly divergent readmission trajectories, with the highest risk among patients with both elevated CRP and CA125, and no clear differences among the other subgroups (Figure 2).

This pattern remained consistent after multivariable adjustment, although the formal interaction term did not reach statistical significance (*p* = 0.080). Compared to the reference group (CRP <20 mg/dl and CA125 < 35 U/ml), patients with CRP ≥20 mg/dl and CA125 ≥ 35 U/ml had a significantly higher risk of readmission (HR: 1.50, 95% CI: 1.07–2.11, *p* = 0.019), while patients with elevated CRP but normal CA125 did not (HR: 0.82, 95% CI: 0.46–1.47, *p* = 0.505).

## Discussion

In this study, we found that the prognostic impact of CRP levels in HF was modified by CA125 levels. Specifically, CRP levels were significantly associated with higher risk of adverse events in patients with elevated CA125 levels (>35 U/ml), whereas it was not if CA125 was <35 U/ml. The underlying mechanism of this interaction remains unclear; however, we hypothesize a biological pathway that connects these processes (19). High CA125 may indicate a clinical state marked by persistent inflammation, heightened vascular permeability, and significant extravascular fluid accumulation. In contrast, elevated CRP without a corresponding increase in CA125 may reflect a primarily systemic inflammatory response, occurring without substantial fluid overload or disruptions in vascular integrity (23–25).

### Pathophysiology

#### High CRP and CA125

In patients with both elevated CRP and CA125, the inflammatory response appears to be closely intertwined with vascular dysfunction and fluid overload (19). The chronic low-grade inflammation observed in HFpEF is driven by several mechanisms, including neurohormonal activation, oxidative stress, and increased levels of pro-inflammatory cytokines (26). These inflammatory mediators not only contribute to myocardial dysfunction but also stimulate serosal cells, leading to an upregulation of CA125 (18).

CRP is a well-established marker of systemic inflammation, produced by hepatocytes in response to cytokines such as IL-6, TNF-α, and IL-1β, all of which are elevated in acute HF (27, 28). In parallel, CA125 is primarily secreted by mesothelial cells in response to mechanical stretch from fluid overload and to inflammatory cytokines (18, 19). In AHF, neurohormonal activation and hemodynamic congestion generate mechanical stress on serosal surfaces (e.g., pleura, pericardium), stimulating CA125 release via pathways such as JNK signaling (18, 19).

Importantly, inflammation and congestion appear to act synergistically. Systemic inflammation increases endothelial permeability, promoting extravascular fluid accumulation, while congestion itself drives local mesothelial stress. This interplay sustains a cycle of inflammation and fluid retention, contributing to worsening HF physiology. The PROMIS-HFpEF study (11) also supports this link: inflammatory biomarkers such as TNFR1, UPAR, IGFBP7, and GDF-15 were associated with both comorbidity burden and echocardiographic dysfunction, underscoring inflammation's central role in HFpEF pathophysiology.

Thus, the coexistence of high CRP and CA125 may reflect a clinical state of persistent inflammation, endothelial dysfunction, and advanced serosal congestion. This combined profile is associated with worse prognosis, capturing a pathophysiological synergy that amplifies disease severity in elderly HFpEF patients (18, 19).

#### High CRP and normal CA125

Patients with elevated CRP but normal CA125 may represent a subset of individuals in which systemic inflammation is present, but the pathophysiological processes that involve volume overload or endothelial dysfunction are not as pronounced. This scenario could point to a more limited role for fluid retention or other inflammatory processes that activate CA125 in these patients. Thus, while CRP may indicate the presence of systemic inflammation and an active immune response, its prognostic value might be less severe without the additional burden of fluid retention, which is captured by CA125.

#### HFpEF phenotype

HFpEF is frequently observed in older patients with multiple comorbidities, which contribute to a chronic inflammatory state at both the systemic and myocardial levels, characterized for a persistently elevated CRP concentrations (18, 20). Moreover, patients with HFpEF often exhibit systemic extravascular congestion, typically secondary to right heart failure, which affects serosal cavities and stimulates mesothelial cells, leading to increased CA125 secretion (18, 20, 26, 29). This interplay between inflammation and volume overload underscores key pathophysiological mechanisms in HFpEF, shaping its clinical phenotype and prognosis.

### Potential clinical implications

This differential impact of CRP levels based on CA125 levels may have important implications for the management of AHF patients. When higher CRP levels coexist with high CA125 the risk increases and potentially the patients require tighter monitoring, and more aggressive diuretic strategies (24, 25). Moreover, patients with elevated CRP and CA125 may benefit from personalized treatment strategies that address both inflammation and volume overload. This could include a combination of intensified diuretic therapy to manage congestion and targeted anti-inflammatory approaches to mitigate systemic inflammation (24, 25, 30). Additional studies are required to elucidate the precise mechanisms driving these findings and to assess their direct clinical implications.

## Limitations

Several limitations must be acknowledged. First, these findings may not be generalizable to heart failure scenarios outside of hospitalization. Secondly, as a retrospective observational study, it cannot establish causality between CRP, CA125, and clinical outcomes. Thirdly, despite adjustments, residual confounding factors (e.g., other inflammatory markers or undiagnosed infections) may have influenced the results. Fourthly, due to limitations in cause-of-death documentation, we used all-cause mortality as the study endpoint. While this approach ensures data completeness, it may underestimate associations specifically driven by cardiovascular or HF-related causes. Fifth, the applicability of our findings to populations beyond elderly, multimorbid patients is limited, and may not be generalizable to younger or less comorbid individuals. Sixthly, only baseline CRP and CA125 levels were analysed; changes over time might provide additional insights into disease progression and prognosis. Finally, full external validation in independent, prospective multicenter cohorts is essential before these findings can be translated into clinical practice.

## Conclusions

In patients with acute heart failure and preserved ejection fraction, the prognostic impact of CRP is influenced by CA125 levels. High CRP levels were associated with higher risk of death or heart failure hospitalization only when coexisted with high CA125. On the contrary, when CA125 was low, high CRP lacked prognostic effect. Further studies are needed to confirm these findings and to understand their pathophysiological significance.

## Data Availability

The data analyzed in this study is subject to the following licenses/restrictions: The dataset analyzed in this study contains sensitive patient health information and is not publicly available due to institutional and ethical restrictions. Access to the dataset may be considered upon reasonable request and subject to approval by the ethics committee of Hospital Universitario Ramón y Cajal. Requests to access these datasets should be directed to paullacer@hotmail.com.
